# Concurrent diabetic ketoacidosis with hyperosmolality and/or severe hyperglycemia in youth with type 2 diabetes

**DOI:** 10.1002/edm2.160

**Published:** 2020-06-15

**Authors:** Jessica Schmitt, AKM. Fazlur Rahman, Ambika Ashraf

**Affiliations:** ^1^ Department of Pediatrics University of Alabama at Birmingham Birmingham AL USA; ^2^ Department of Biostatistics University of Alabama at Birmingham Birmingham AL USA

**Keywords:** acute kidney injury, adolescent, altered mental status, diabetes mellitus, type 2, diabetic ketoacidosis, hyperglycaemic hyperosmolar nonketotic coma

## Abstract

**Introduction:**

Prevalence of diabetic ketoacidosis (DKA) complicated by severe hyperglycaemia and hyperosmolality and its outcomes in youth with type 2 diabetes (T2DM) are not well‐described. Our aim is to determine the frequency and clinical outcomes of isolated DKA, DKA with severe hyperglycaemia (DKA + SHG) and DKA with hyperglycaemia and hyperosmolality (DKA + HH) in youth with T2DM admitted for acute hyperglycaemic crisis.

**Methods:**

Through retrospective medical record review, patients with T2DM were identified and categorized into isolated DKA, DKA + SHG (DKA + glucose ≥33.3 mmol/L) and DKA + HH (DKA + glucose ≥33.3 mmol/L + osmolality ≥320 mmol/kg).

**Results:**

Forty‐eight admissions in 43 patients ages 9‐18 were included: 28 (58%) had isolated DKA, six (13%) had DKA + SHG and 14 (29%) had DKA + HH. Subgroups’ demographics and medical history were similar. Seventeen patients (35%) had acute kidney injury (AKI). Odds of AKI were higher in DKA + SHG and DKA + HH relative to isolated DKA (*P* = .015 and .002 respectively). Frequency of altered mental status (AMS) was similar among groups. Three patients (6%) had concurrent soft‐tissue infections at presentation with no differences among subgroup. Three patients (6%) had other medical complications. These occurred only in patients with AKI and DKA + SHG or AKI and DKA + HH.

**Conclusions:**

In youth with T2DM, severe hyperglycaemia ± hyperosmolality frequently complicates DKA. Youth with DKA and features of hyperglycaemic hyperosmolar syndrome, including isolated severe hyperglycaemia, have increased odds of AKI.

## Graphical abstract

Youth with type 2 diabetes who have diabetic ketoacidosis often have features of hyperosmolality and/or severe hyperglycaemia. These features are associated with increased odds of acute kidney injury.

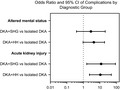

## 1 INTRODUCTION

As prevalence of type 2 diabetes (T2DM) rises among America's youth,^1^ paediatric providers will be increasingly responsible for treating acute hyperglycaemic complications of T2DM, including diabetic ketoacidosis (DKA) and hyperglycaemic hyperosmolar syndrome (HHS). In DKA, insulin deficiency is severe enough that in addition to hyperglycaemia, lipolysis begins and ketosis develops.^2^ It is defined by the presence of hyperglycaemia with a serum blood glucose of > 11 mmol/L (approximately 200 mg/dL), acidosis with a pH less than 7.3 and/or a bicarbonate of < 15 mmol/L and ketosis.^3^, ^4^ In HHS there is enough insulin activity to prevent ketosis, but insufficient insulin activity to prevent hyperglycaemia.^2^ HHS is characterized by minimal acidosis with a venous pH > 7.25 and serum bicarbonate > 15 mmol/L, minimal to no ketonuria, severe hyperglycaemia with a serum blood glucose > 33.3 mmol/L (>600 mg/dL) and hyperosmolality with serum osmolality of > 320 mmol/kg.^4^

Previously considered two distinct entities, in clinical practice the conditions overlap significantly.^5^, ^6^, ^7^ A mixed presentation of DKA complicated by severe hyperglycaemia and hyperosmolality is common in patients hospitalized with acute hyperglycaemic crisis, occurring in up to 14% of children^8^ and 27% of adults.^6^

Retrospective cohort studies and case reports report high rates of mortality, altered mental status (AMS), acute kidney injury (AKI), thrombosis, rhabdomyolysis and other medical complications in those with mixed DKA/HHS and isolated HHS when compared to isolated DKA.^5^, ^6^, ^8^, ^9^ Persistent hyperglycaemia and osmotic diuresis lead to progressive volume depletion. Pre‐renal injury from dehydration causes a decline in glomerular filtration rate, reducing renal glucose clearance, exacerbating hyperglycaemia and contributing to hyperosmolality.^10^ A recent large retrospective case series in adults showed a higher rate of mortality in patients with mixed DKA/HHS compared to isolated DKA and isolated HHS.^6^

Children's of Alabama, a tertiary care university children's hospital in the southeastern United States where T2DM prevalence is high,^11^ is uniquely positioned to review complications and frequency of isolated DKA and DKA complicated by severe hyperglycaemia and/or hyperosmolality in youth with T2DM. We therefore aimed to determine how frequently DKA is complicated by severe hyperglycaemia and/or hyperosmolality in paediatric patients admitted for acute hyperglycaemic complications of T2DM. Our secondary aim was to evaluate the frequency of acute medical complications, including AKI and AMS, in this patient cohort. Finally, we aimed to identify any risk factors associated with hyperosmolality.

## 2 METHODS

### 2.1 Data source

A retrospective electronic medical record review was conducted. Initial query identified admissions to the intensive care unit (ICU) or step‐down ICU with International Classification of Disease 10 diagnosis code of diabetes (E8.xx, E10.xx, E11.xx or E13.xx) or hyperglycaemia (R73.9) and who were admitted from January 2013 through December 2018. Inclusion criteria were age 0‐20 years, admission to the ICU or step‐down ICU, and diagnosis of T2DM. Exclusion criteria included steroid‐induced diabetes, type 1 diabetes mellitus, and insufficient data to confirm diagnosis of T2DM. Diagnosis of T2DM was confirmed based on documented clinical assessment, negative autoantibodies when measured, and elevated c‐peptide if autoantibodies were not assessed. Investigator JS reviewed individual medical records at diagnosis and follow‐up to ensure accuracy of T2DM diagnosis. For patients with multiple admissions, each admission was evaluated as a unique event.

### 2.2 Variables

Cases were identified as belonging to one of three possible diagnostic subgroups: isolated DKA, DKA with severe hyperglycaemia (DKA + SHG) or DKA with severe hyperglycaemia and hyperosmolality (DKA + HH). Isolated DKA was defined by presence of hyperglycaemia with a serum blood glucose of 11 to 33.2 mmol/L (approximately 200 to 599 mg/dL), acidosis with a venous pH of less than 7.3 or a bicarbonate of < 15 mmol/L.^3^, ^4^ As serum and urine ketones may not be obtained at presentation, ketone measurement was not an inclusion criterion.

Subjects with DKA + SHG met criteria for DKA, but also had a blood glucose at presentation that was > 33.3 mmol/L (600 mg/dL). Subjects with DKA + HH were those with DKA complicated by both severe hyperglycaemia and hyperosmolality with a serum osmolality of > 320 mmol/kg at presentation. When serum osmolality was not measured, it was calculated based upon the calculation serum osmolality (mmol/kg) =^12^:

$$2\left(\text{serum}\;\text{sodium}\;\text{in}\;\text{mmol}/L\right)+\frac{\text{glucose}\;\text{inmgperdL}}{18}+\frac{\text{bloodureanitrogenin}\text{mgperdL}}{2.8}$$

Data collected included patient demographics, personal and family medical history, hospital course and laboratory and clinical assessment at presentation. Corrected sodium for hyperglycaemia was calculated based upon a 1.6 mmol/L increase in sodium for every 5.55 mmol/L (100 mg/dL) serum glucose was above 5.55 mmol/L (100 mg/dL).^2^Presence or absence of altered mental status (AMS) was determined by manual chart review of documented physician assessment and neurologic exam.. A positive psychiatric history was defined as any documented psychiatric disorder except for attention deficit hyperactivity disorder. AKI was defined the 2012 clinical practice guideline for AKI as a creatinine 1.5x above baseline.^13^ For patients without a known baseline creatinine, the discharge creatinine was used as a proxy. Other medical complications were determined by manual chart review.

### 2.3 Statistical analysis

Mean and standard deviation (SD) are reported for normally distributed continuous variables with paired comparisons done with Student *t* tests. Median and interquartile range (IQR) are reported for skewed continuous variables with paired comparisons by Mann‐Whitney *U* test. For comparing the three diagnostic subgroups, ANOVA and Kruskal‐Wallis tests were used to assess for differences among normally distributed and skewed continuous variables, respectively. Categorical variables are summarized by counts and percentage with analysis by chi‐squared test or Fisher exact test as appropriate. Linear association among continuous variables was assessed with Pearson's correlation. We performed logistic regression to identify the potential risk factors of the outcomes: diagnostic subgroups, AKI and AMS. To quantify the effects of association, we estimated odds ratio with their 95% confidence intervals. Prior to multivariate logistic regression, we performed a stepwise initial variable selection and variables with p‐value less than or equal to 0.15 were initially included in the multivariate models. All hypothesis tests were two‐tailed, using a *P*‐value < .05 to indicate statistical significance. We performed analyses in SAS for windows version 9.4 (SAS Institute).

### 2.4 Ethics

This study was exempted by the University of Alabama at Birmingham Internal Review Board.

## 3 RESULTS

### 3.1 Patient characteristics

**Figure 1:**
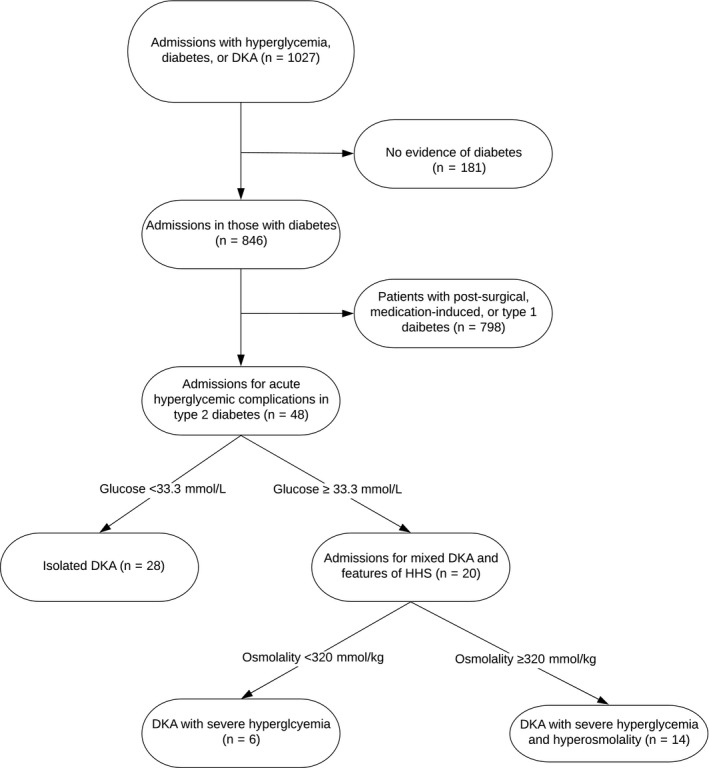
Flow diagram of medical record review. DKA, diabetic ketoacidosis; HHS, hyperglycaemic hyperosmolar syndrome

There were 1027 patients identified after initial query of the medical record, with 48 admissions in subjects age between 9 and 18 meeting inclusion criteria. Figure 1. On average, patients were non‐Hispanic, African‐American, male, governmentally insured, adolescents with obesity. Developmental delay was uncommon, but a positive psychiatric history was present in 22%. A family history of diabetes was universal. Insulin resistance was already diagnosed in 38% of patients, with 25% of those prescribed insulin prior to admission. Table 1. No patients were treated with sodium glucose cotransporter 2 inhibitors. Three patients had multiple admissions. One had three isolated DKA admissions,one had two isolated DKA admissions. The final patient had three admissions. The first was DKA + SHG with AKI and a soft‐tissue infection, the second and third admission where isolated DKA without AKI, infection or complications.

**Table 1:** Baseline characteristics and laboratory assessment

	Isolated DKA (n = 28)	DKA + SHG (n = 6)	DKA + HH (n = 14)	*P*‐value
Male	16 (57)	4 (67)	7 (50)	.84
African‐American	25 (89)	4 (67)	13 (93)	.29
Non‐Hispanic	25 (89)	5 (83)	14 (100)	.32
Positive psychiatric history (n = 46)	6 (23) n = 26	3 (50)	1 (7)	.10
Developmental Delay	4 (14)	0 (0)	1 (7)	.83
History of pre‐diabetes or type 2 diabetes	12 (43)	3 (50)	3 (21)	.36
History of insulin use	10 (36)	2 (33)	0 (0)	.020
Weight (kg)^a^	92.1 (76.8‐112.4)	123.9 (93.6‐149.0)	92.8 (76.3‐110)	.22
BMI *z*‐score (n = 45)	2.25 (0.52) n = 26	2.58 (0.37)	2.34 (0.66)	.42
Age at admission (years)	15.4 (1.8)	15.8 (1.4)	14.0 (2.7)	.086
Age at diagnosis of type 2 diabetes (years) (n = 47)	13.7 (2.7)	14.3 (2.9)	13.5 (2.4)	.82
Private insurance	5 (17)	0 (0)	3 (21)	.74
Corrected sodium (mmol/L)^a^	141 (139‐144)	141 (136‐148)	157 (151‐160)	<.001
Potassium (mmol/L)	4.3 (0.9)	4.4 (1.1)	5.0 (0.9)	.080
Phosphorus (mmol/L)^a^	1.10 (0.81‐1.26)	1.03 (0.84‐1.26)	1.29 (0.90‐1.39)	.38
pH^a^	7.20 (7.10‐7.23) n = 27	7.14 (7.07‐7.17)	7.17 (7.07‐7.24)	.67
Bicarbonate (mmol/L)^a^	7.0 (4.9‐11.0)	7.0 (6.0‐8.0)	8.0 (6.0‐12.0)	.65
Blood glucose (mmol/L) [mg/dL]	23.0 (6.9) [414 (125)]	45.5 (7.3) [820 (132)]	54.8 (11.3) [987 (204)]	<.001
Haemoglobin A1c (mmol/mol) [%]^a^	130 (113‐130) [14.0 (12.5‐14.0)]	113 (92‐121) [12.5 (10.6‐13.2)]	130 (109‐130) [14.0 (12.1‐14.0)]	.21
Creatinine (µmol/L)^a^	61.9 (53.0‐79.6)	88.4 (79.6‐123.8)	97.2 (79.6‐168.0	<.001
Haematocrit (%)	46.0 (5.8) n = 27	52.0 (8.3)	50.0 (7.4)	.079
Calculated osmolality (mmol/kg)	299.0 (7.5)	304.6 (9.4)	341.6 (16.9)	<.001
Urine ketones^a^	2 (2‐4) n = 21	2.5 (2‐3)	3 (1‐3)	.66

Note

Data are n (%) or mean (SD) except where otherwise noted.

Abbreviations: DKA, diabetic ketoacidosis; DKAwHG, DKA with severe hyperglycaemia; DKA + HHS, DKA with hyperosmolality and hyperglycaemia.

^a^ Median (IQR) shown for skewed variable.

### 3.2 Subgroup analysis

Of the 48 admissions, 28 (58%) met criteria for isolated DKA, 6 (13%) for DKA + SHG, and 14 (29%) for DKA + HH. Median duration of hospital admission for isolated DKA was 65.1 hours (IQR 45.9‐87.1), 104.5 hours (IQR 68.1‐185.9) for DKA + SHG, and 73.1 hours (IQR 66.5‐95.9) for DKA + HH (*P* = .048). Regarding demographics and prior medical history, on unadjusted analysis, only frequency of prior insulin use differed among the three diagnostic subgroups (*P* = .020). On adjusted analysis, there were no significant differences in demographic data or medical history between DKA + SHG and isolated DKA. Adjusted analysis of those with DKA + HH and isolated DKA found that younger age was independently associated with DKA + HH over isolated DKA (OR 0.48, 95% CI 0.27‐0.84, *P* = .010).

Measured pH and bicarbonate did not differ significantly among groups. Of those with isolated DKA, 11 had severe DKA with a pH < 7.1 or bicarbonate < 5 mmol/L, 10 had moderate DKA with pH of 7.1‐7.2 or bicarbonate 5‐9 mmol/L, and seven had mild DKA with a pH > 7.2 and bicarbonate > 10 mmol/L. For those with DKA + SHG, two had severe DKA, three had moderate DKA, and one had mild DKA. In the DKA + HH group, five patients had severe DKA, five had moderate DKA, and four had mild DKA.

Adjusted analysis found higher creatinine remained independently associated with increased odds of DKA + HH over isolated DKA (adjusted OR 148.2, 95% CI 2.4‐9139.2, *P* = .017). This association was not seen when comparing those with DKA + SHG and isolated DKA (adjusted OR 28.73, 95% CI 0.86‐958.66, *P* = .061).

**Figure 2:**
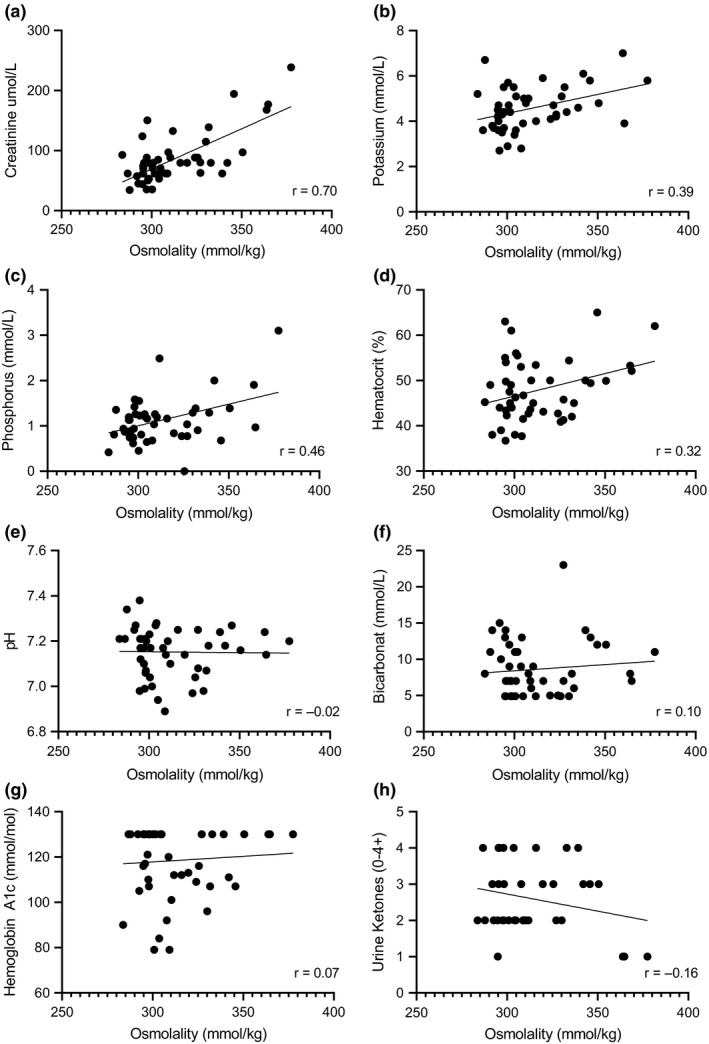
Correlation plots of serum osmolality and other laboratory analysis at presentation. (A‐D) There is a positive linear association between osmolality and creatinine, potassium, phosphorus and haematocrit (*P* < .05 for all). (E‐H) There is no linear association between osmolality and pH, bicarbonate, haemoglobin A1c nor urine ketones (*P* > .05)

Mean osmolality (mmol/kg) was 299.0 ± 7.5 for those with isolated DKA, 304.6 ± 9.4 for those with DKAwHG, and 341.6 ± 14.9 for those with DKA + HH (*P* < .001). Serum osmolality correlates positively with creatinine, potassium, phosphorus and haematocrit. No linear association was found between serum osmolality and pH, bicarbonate, haemoglobin A1c or urine ketones. Figure 2.

### 3.3 Altered mental status

In 47 of the 48 admissions, there was a clearly documented assessment of mental status. AMS was present in twelve admissions (26%). Odds of AMS were similar between diagnostic subgroups. For DKA + SHG, unadjusted OR of AMS relative to isolated DKA was 2.88 with a 95% CI of 0.39‐21.29 (*P* = .30). For DKA + HH, unadjusted OR of AMS relative to isolated DKA was 4.31 with a 95% CI of 0.96‐19.31 (*P* = .056). See Figure 1. Patients with AMS had a median pH of 7.07 (IQR 6.97‐7.18) and those without AMS had a median pH of 7.20 (IQR 7.14‐7.25) (*P* = .003). On multivariate analysis, pH remained independently associated with AMS with an adjusted OR of 0.07, 95% CI: 0.01‐0.63, *P* = .019 for 1 standard deviation (0.11 unit) change from mean pH 7.15. Patients with AMS had a median phosphorus of 1.39 mmol/L (0.94‐2.00 mmol/L) (4.3 mg/dL IQR: 2.9‐6.2 mg/dL) while those without had a median phosphorus of 0.97 mmol/L (0.81‐1.26) (3.0 mg/dL IQR: 2.5‐3.9) (*P* = .0368). On multivariate analysis, phosphorus remained independently associated with AMS with an adjusted OR of 6.54, 95% CI: 1.31‐32.72 (*P* = .022).

### 3.4 Acute kidney injury

**Figure 3:**
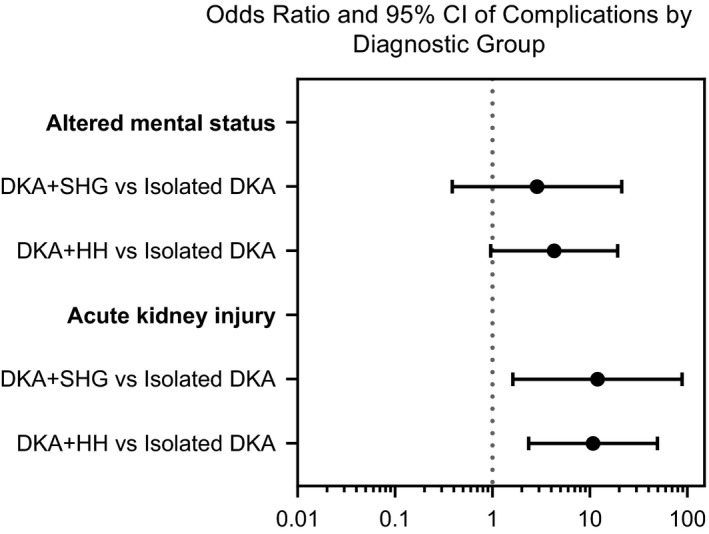
Odds ratio (OR) of altered mental status and acute kidney injury with 95% confidence interval (CI) by diagnostic group. DKA, diabetic ketoacidosis; DKA + SHG, DKA with severe hyperglycaemia; DKA + HH, DKA with severe hyperglycaemia and hyperosmolality

Seventeen patients (35%) had AKI. At admission, AKI was present in four patients (14%) with isolated DKA, three patients (50%) with DKA + SHG, and nine patients (64%) with DKA + HH (*P* = .001). One patient who had three admissions had AKI during the first admission, but not in the subsequent admissions. Unadjusted odds of AKI were 12.0 times higher in those with DKA + SHG compared to isolated DKA with a 95% CI of 1.6‐88.7 (*P* = .015). Unadjusted odds of AKI were 10.8 times higher in those with DKA + HH compared to isolated DKA with a 95% CI of 2.4‐49.5 (*P* = .002, Figure 3. AMS occurred in seven patients (44%) with AKI and five (16%) of those without AKI (*P* = .075). Other medical complications occurred in three patients (6%) with AKI and 0 patients (0%) without AKI (*P* = .039).

AKI was more common in males (76% vs 45%, *P* = .037), those with higher BMI z‐score (2.58 ± 0.49 vs 2.17 ± 0.54 *P* = .016) and those with an older age at diagnosis of pre‐diabetes or overt T2DM (15.1 ± 2.0 vs 13.0 ± 2.6, *P* = .007). Mean osmolality of those with AKI was 327.3 ± 27.0 mmol/kg and 303.8 ± 13.2 mmol/kg in those without AKI (*P* < .001). On multivariate analysis, a positive prior psychiatric history (adjusted OR 112.4, 95% CI 2.9‐4345.0, *P* = .011), older age at diagnosis of pre‐diabetes/T2DM (adjusted OR 2.07, 95% CI 1.11‐3.84, *P* = .022), and higher osmolality (adjusted OR 1.13, 95% CI 1.04‐1.22, *P* = .004) were independently associated with increased odds of AKI.

### 3.5 Concurrent infection

Three patients had soft‐tissue infections at presentation. Patients were not on insulin pumps, and infections were not associated with insulin injection sites. Two patients with isolated DKA had soft‐tissue infections. One had an abscess was on the buttock, the other had a labial pustule; both were complicated by surrounding cellulitis. They were treated surgical drainage and systemic antibiotics. One patient with DKA + SHG had cellulitis of the mons pubis that required oral antibiotics. The frequency of infection was 67% in isolated DKA, 33% in DKA + SHG and 0% in DKA + HH (*P* = .22).

### 3.6 Other medical complications

Three patients had medical complications other than soft‐tissue infections, AKI or AMS. One patient with DKA + SHG required intubation due to severity of AMS and suffered a bladder haematoma. Another patient with DKA + SHG had pancreatitis with triglycerides of 1014 mg/dL. One patient with DKA + HH had rhabdomyolysis, parotiditis, a deep vein thrombosis and remained on renal replacement with dialysis at discharge. Rate of complications was 0% in isolated DKA was 0%, 67% in DKA + SHG, and in 33% in DKA + HH (*P* = .013).

## 4 DISCUSSION

Traditionally taught as two distinct entities, DKA and HHS represent two ends of a continuous spectrum. In this retrospective review of a large number of youth with T2DM, we report a high rate of severe range hyperglycaemia and hyperosmolality in patients admitted for ‘DKA’. Our work adds to existing case reports of patients who present with mixed features of DKA and HHS^5^, ^6^, ^8^, ^14^, ^15^ but is unique in exclusion of patients with type 1 diabetes and adults. To our knowledge, this is the first time a comparison of three distinct diagnostic subgroups, isolated DKA, DKA + SHG and DKA + HH in youth with T2DM has been reported.

In youth with T2DM hospitalized for acute hyperglycaemic crisis, relative to previous reports,^6^, ^8^ we found a high rate of severe hyperglycaemia and hyperosmolality with one or both of these occurring in 42% of patients. Demographics and medical history, including developmental delay and psychiatric history, were similar among patients in the diagnostic subgroups. This counters previous case reports in youth with diabetes complicated by hyperosmolality in which high rates of cognitive impairment and mental health disorders have been reported.^14^, ^16^, ^17^ Pre‐diabetes/T2DM had been previously diagnosed in a large proportion of our cohort, suggesting that a proportion of admissions for isolated DKA, DKA + SHG and DKA + HH in this population could be preventable with increased education, awareness of symptoms of decompensated diabetes, and aggressive insulin administration at home.

Interestingly, severity of acidosis as measured by pH and bicarbonate was similar among groups. Serum ketones are not routinely measured at this institution, but urine ketones among the three groups showed no difference in severity of ketosis. Presence of acidosis and ketosis is not protective against hyperosmolality in youth with T2DM. The similar severity of ketosis among all groups supports the notion of DKA and HHS as two ends of a clinical spectrum rather than discrete conditions in youth with T2DM. While we found worsening acidosis as measured by pH to be associated with odds of AMS, increasing osmolality and diagnostic subgroup were not correlated with increased rates of AMS. Lack of association may be related to the relatively few number (n = 12) of patients in our cohort affected by AMS.

Osmolality was found to correlate strongly and positively with creatinine, and overt AKI was higher in those with DKA + SHG and DKA + HH relative to isolated DKA. This likely reflects the importance of renal clearance of glucose to prevent hyperosmolality. As the glomerular filtration rate decreases, the kidneys ability to excrete glucose declines, leading to worsening of hyperglycaemia.^10^ Osmolality was also found to positively correlate with haematocrit, again suggesting a larger degree of intravascular volume depletion in those with hyperosmolality.

Concurrent soft‐tissue infection was seen in three patients (6%), with no significant difference in frequency of infection among diagnostic subgroup. Other medical complications were seen in 3 patients (6%) and the differences among subgroup reached statistical significance. Although the total number of complications was low, only patients with DKA + SHG and DKA + HH and AKI were affected. This suggests that features of HHS in addition to AKI are risk factors for severe complications in patients with T2DM and DKA.

Our study has several limitations. We had no patients with isolated HHS, limiting ability to compare outcomes in those with DKA + HH and DKA + SHG compared to isolated HHS. Additionally, we had a small number of patients with DKA + SHG, which likely affected ability to distinguish between small differences among the groups. While we could have included those with DKA + SHG with the isolated DKA group, we felt that the severity of hyperglycaemia was indicative of progressive renal injury,^10^ increased duration of illness, and progression towards hyperosmolality and warranted individual assessment. Finally, analysis was dependent upon clinical documentation and available laboratory data and some subjects lacked complete data for analysis. There were three patients with multiple admissions in our cohort. We elected to treat each admission as a unique encounter since our primary goal was to determine how often patients with DKA and T2DM present with features of HHS. Additionally, if we did not count each admission individually, we would have decreased the likelihood of identifying patient characteristics that increase risk of isolated DKA, DKA + SHG or DKA + HH.

Despite these limitations, our work adds to the growing body of literature on mixed DKA + HHS in patients with diabetes and is one of the larger studies specific to youth with T2DM. Further work is needed to study risks of developing mixed DKA/HHS in youth with T2DM and intervention studies are needed to identify ways to prevent complications of this increasingly common condition.

## CONFLICT OF INTEREST

Authors have no conflicts of interest to declare.

## AUTHOR CONTRIBUTION

JS conceptualized and designed the study, acquired the data, assisted with data analysis, drafted the initial manuscript and approved the final manuscript. AKMFR performed data analysis and reviewed the final manuscript. AA assisted with study design and critically revised the manuscript. All authors read and approved the final manuscript.

## Data Availability

The data supporting the findings of this study are not publicly available due to ethical considerations and inclusion of protected health information.
